# Development of Chitosan-Based Composite Films Incorporating Anchovy Byproduct Hydrolysates

**DOI:** 10.3390/polym17131754

**Published:** 2025-06-25

**Authors:** Bilge Bilgin Fıçıcılar, Koray Korkmaz

**Affiliations:** Fatsa Faculty of Marine Sciences, Fisheries Technologies Engineering, Ordu University, Fatsa, 52200 Ordu, Turkey; bilgebilginficicilar@odu.edu.tr

**Keywords:** chitosan, films, fish protein hydrolysate, anchovy byproduct, waste valorization

## Abstract

This study developed edible composite films incorporating the anchovy (*Engraulis encrasicolus*) byproduct protein hydrolysate (ABPH) into a chitosan matrix and evaluated their physicochemical, structural, and functional properties for food packaging applications. ABPH, produced by Flavourzyme enzymatic hydrolysis, exhibited high hydrolysis (54–57%) and high protein content (80.7 ± 0.94%). Films were produced using 1%, 2%, and 3% ABPH (CH-FP1, CH-FP2, and CH-FP3) by the casting method. Characterization of the films revealed that a higher ABPH concentration increased water swelling, solubility, and opacity, while tensile strength decreased and elongation at break improved, indicating greater flexibility. FTIR analysis showed that ABPH was incorporated through enlarged amide I and II bands and broader -OH/NH regions, suggesting hydrogen bonding and protein–polysaccharide interactions. SEM images demonstrated good dispersion at low concentrations and more uniform surfaces at higher ABPH levels. This suggests that chitosan–ABPH composite films can serve as biodegradable, protein-enriched packaging materials with adjustable mechanical and barrier properties to valorize fishery waste and sustainable food packaging solutions.

## 1. Introduction

Chitosan, a natural polysaccharide derived from the deacetylation of chitin, has gathered considerable attention in biomedical, pharmaceutical, and food packaging research due to its film-forming capacity, biodegradability, biocompatibility, and antimicrobial properties [1,2]. These properties make chitosan a compelling candidate for the production of edible and biodegradable films as alternatives to conventional synthetic polymers. However, films composed of pure chitosan often exhibit limited mechanical strength, reduced flexibility, and low antioxidant capacity, which may restrict their broader application in food packaging [3]. To overcome these limitations, researchers have increasingly explored the incorporation of functional and bioactive compounds, particularly those derived from proteins, as a practical and effective way to enhance the overall performance of chitosan-based films. Fish protein hydrolysates (FPHs) are bioactive compounds produced by enzymatic hydrolysis of fish proteins into smaller peptides and amino acids, which significantly enhances their solubility, digestibility, and functional attributes such as antioxidant, antimicrobial, and emulsifying activities. Typically sourced from fish processing byproducts like heads, bones, and viscera, FPHs represent a sustainable solution for waste valorization. Due to their biofunctional properties and compatibility with biopolymers, FPHs have been widely incorporated into biodegradable films and coatings to improve their mechanical integrity and oxidative stability. Notably, their integration into chitosan matrices has been shown to synergistically enhance the mechanical, antioxidant, and antimicrobial performance of composite biopolymer films [4,5,6].

In parallel, the efficient valorization of seafood processing byproducts has become increasingly important to reduce waste and support circular bioeconomy initiatives. It is estimated that nearly 35% of global seafood production results in discarded byproducts, including heads, bones, viscera, and skin [7]. Anchovy (*Engraulis encrasicolus*), a small pelagic fish with widespread distribution in the East Central Atlantic, Mediterranean, and Black Sea, is a prominent species in both European and Turkish fisheries. In Turkey alone, annual landings increased from 125,980 tons in 2022 to 273,914 tons in 2023 [8]. Filleting anchovy typically generates approximately 32% waste [9], which includes parts rich in protein and minerals. These byproducts, often discarded or underutilized, can be enzymatically hydrolyzed to produce fish protein hydrolysates (FPHs), which exhibit improved solubility, bioavailability, and bioactivity compared to intact proteins [10]. Previous studies have shown that FPHs possess antioxidant and antihypertensive effects and offer functional advantages for film-forming applications, such as network formation, flexibility, and barrier properties [11,12].

Although some studies have examined the incorporation of protein hydrolysates into biodegradable films, their application in chitosan-based matrices is still limited. Gómez-Estaca, et al. [13] developed composite edible films combining fish gelatin and chitosan and demonstrated enhanced antimicrobial activity, highlighting the potential of protein-rich marine sources for active packaging. Kchaou, et al. [14] incorporated cuttlefish protein hydrolysates into gelatin-based films and reported improvements in antioxidant properties and UV-barrier capacity, though accompanied by reduced tensile strength and flexibility. Similarly, Santos, et al. [15] used protein hydrolysates derived from free-range chicken feathers in keratin-based films and observed enhanced antioxidant activity and biodegradability, alongside notable changes in solubility and mechanical properties. These studies demonstrate the functional benefits of incorporating protein hydrolysate into biopolymer films, particularly in terms of their antioxidant and active properties. However, their interaction with chitosan matrices and influence on the physicochemical structure of such films require further exploration. The present study aims to address this gap by developing edible composite films based on chitosan and incorporating ABPH at three concentrations (1%, 2%, and 3% *w*/*v*). The films were prepared via the casting method and characterized for their physicochemical, structural, and mechanical properties. By evaluating the impact of ABPH addition on the properties of the films, this work contributes to the development of functional packaging materials and supports the valorization of anchovy processing waste into high-value applications.

To the best of our knowledge, this is the first study to incorporate enzymatically derived anchovy byproduct protein hydrolysate (ABPH) into chitosan-based films for packaging applications. By evaluating the effects of ABPH at different concentrations on film performance, this work introduces a novel valorization approach for anchovy waste and contributes new insights into chitosan–protein hydrolysate interactions.

## 2. Materials and Methods

### 2.1. Materials

Anchovy (*Engraulis encrasicolus*), a species commonly found in the Black Sea region, was used in this study, specifically its viscera, head, and bones. Byproducts were obtained from freshly processed fish at the facility, placed in foam boxes with ice, and transported to the laboratories of Ordu University, Fatsa Faculty of Marine Sciences. Until further processing to obtain protein hydrolysate, the samples were stored at −40 °C.

Chitosan, sourced from Chitoglobal Biyoteknoloji, had the following characteristics: a semi-flakes form with a 10-mesh size, 50.000 molecular weight, a bulk density of 0.31 g/mL, a pH of 7, an ash content of 0.7%, and a moisture content of 5%. It had a viscosity of 50 mPas at 23 °C and a deacetylation degree of 97%. Flavourzyme, a commercial enzyme, was sourced from Novozymes A/S (Bagsvaerd, Denmark). All other chemicals used in this study were commercially available and of analytical grade.

### 2.2. Anchovy Byproduct Protein Hydrolysate Preparation

Frozen anchovy head, viscera, and bones stored in 5 kg packages were thawed at room temperature and processed using a mincing machine (Empero, EMP.12.01.P, Konya Turkey). The minced byproducts were heated in a water bath (Memmert, WNB 22, Schwabach, Germany) at 90 °C for 20 min to inactivate endogenous enzymes. After cooling, distilled water was added at a 1:1 ratio, and the mixture was homogenized using an ultra-turrax (IKA T25, Staufen, Germany).

Flavourzyme, a proteolytic enzyme complex from Aspergillus oryzae with both endo- and exopeptidase activities, was used for hydrolysis at the optimum pH of 7.0, enabling the efficient breakdown of fish proteins into bioactive peptides and amino acids that enhance the functional properties of the hydrolysates. Hydrolysis was performed at 1% enzyme, 1.5 h, and 50 °C. Afterwards, Flavourzyme was inactivated at 90 °C for 5 min before cooling for 15 min. Following hydrolysis, the solution was centrifuged (Sigma 3-30KS, Osterode, Germany) at 2067× *g* for 20 min to separate the phases. The liquid phase was freeze-dried, and the resulting protein hydrolysates were stored at 4 °C until analysis.

### 2.3. Degree of Hydrolysis (%DH)

The degree of hydrolysis (%DH) was determined based on the method described by Hoyle and Merritt [16], which measures the proportion of protein that dissolves in trichloroacetic acid (TCA) relative to the total protein content after hydrolysis.

To conduct the analysis, the hydrolysate was mixed with 20% TCA in a 1:1 ratio and centrifuged at 15,000× *g* for 20 min at 4 °C. The soluble proteins in the clear supernatant (10% TCA solution) were then quantified.

The degree of hydrolysis was calculated according to Equation (1):

%DH = (N_o_/N_t_) × 100 (1)

where N_o_ = amount of protein soluble in 10% TCA, and N_t_ = total protein content in the hydrolysate.

### 2.4. Chemical Composition

The chemical compositions of the raw material and the hydrolysate were assessed as follows. The moisture content was measured using the gravimetric method [17], where samples were dried in an oven at 105 °C until reaching a constant weight. The protein content was determined using the Kjeldahl method [18] with a nitrogen conversion factor of 6.25 to estimate the total protein. The lipid content was analyzed using the Soxhlet extraction method [19]. The ash content was measured in a muffle furnace at 500–600 °C following the gravimetric method [20].

### 2.5. Amino Acid Composition of ABPH

The total amino acid content in the ABPH was determined using a modified version of the methods employed by Lee and Hwang [21] and Chan and Matanjun [22] on an LC-MS/MS (Thermofisher Scientific Inc., Walthram, MA, USA) instrument. According to this method, 0.2 g of homogenized sample was weighed into a solution containing 10 mL of 6 N HCl. After tightly sealing the mixture, the test tube was vortexed for 5 min and then placed in an oven at 110 °C for 24 h to complete the hydrolysis. The mixture was then cooled to room temperature and centrifuged at 4000 rpm for 15 min at 4 °C. The supernatant obtained after centrifugation was filtered through a 0.45 μm PTFE membrane filter and injected into the LC-MS/MS instrument for analysis.

### 2.6. Preparation of Films

The films were prepared using the casting method [19]. A 2% (*w*/*v*) chitosan solution and ABPH at concentrations of 1%, 2%, and 3% (*w*/*v*) were separately dissolved in 0.1 M acetic acid and mixed thoroughly by continuous mechanical stirring overnight at room temperature. To enhance the flexibility of the films, 2.5% (*w*/*v*) glycerol was incorporated into all formulations as a plasticizing agent. The prepared film-forming solutions were centrifuged at 13,640× *g* for 10 min using a Hermle Labortechnik Z 32 HK (Wehingen, Baden-Württemberg, Germany) centrifuge to remove undissolved particles. After centrifugation, 30 mL of each solution was poured into Petri dishes and left to dry at 25 °C for 1 to 3 days under controlled conditions to ensure uniform film formation.

Four film samples were produced and named as follows:

CH-FPH: 2% chitosan;

CH-FPH1: 2% chitosan + 1% ABPH;

CH-FPH2: 2% chitosan + 2% ABPH;

CH-FPH3: 2% chitosan + 3% ABPH.

### 2.7. Film Characterization

#### 2.7.1. Moisture Content Analysis

The moisture content of the films was determined using a moisture analyzer (AND MX-50, Tokyo, Japan). Approximately 1 g of each film sample was placed in the analyzer and dried at 105 °C, with the final moisture content recorded as a percentage.

#### 2.7.2. Thickness Measurement

The thickness of the films was measured using a Digimatic Micrometer. Measurements were taken at five randomly selected points for each film to ensure accuracy and consistency.

#### 2.7.3. Water Swelling

The swelling index of the films was determined according to [20]. Pre-dried film samples were weighed using a precision balance. The films were placed in beakers in 50 mL of distilled water and shaken continuously at 25 °C and 100 rpm for 30 min using a shaker (SSL1, Stuart, UK). The swelling ratio was expressed as the ratio of g water uptake to g total film solids.

#### 2.7.4. Color Measurement

The color of the films was assessed using a Konica Minolta CM-5 Colorimeter (Osaka, Japan), which recorded the L* (lightness), a* (red–green spectrum), and b* (blue–yellow spectrum) values. The total color difference (Δ*E*) between the control film (CH) and the other film samples was calculated using the CIELAB standard equation:

$$\Delta E=\sqrt{\left.{\left(L_{2}-L_{1}\right)}^{2}+{\left(a_{2}-a_{1}\right)}^{2}+{\left(b_{2}-b_{1}\right)}^{2}\right.}$$

Reference color (CH): L* = 33.50, *a* = 0.03, *b* = −0.37.

#### 2.7.5. Opacity Analysis

The opacity of the films was determined using a UV-1280 UV–VIS Spectrophotometer (Shimadzu, Kyoto, Japan). The opacity was calculated by dividing the film thickness (mm) by its absorbance at 600 nm, providing insight into the material’s transparency.

#### 2.7.6. Water Solubility

To evaluate the water solubility of the films, small 2 × 2 cm strips were cut and weighed to determine their initial dry weight. These strips were then dried in an oven at 100 °C for 24 h, and their final dry weight was recorded. Next, the dried films were immersed in 15 mL of distilled water at room temperature and stirred for 24 h. After this period, the undissolved portion was separated using Whatman filter paper and dried again to measure the remaining dry weight. The water solubility percentage (WS%) was calculated according to Equation (2).

WS% = (Initial dried weight (mg) − Final dried weight (mg))/(Initial dried weight (mg)) × 100(2)

#### 2.7.7. Mechanical Properties of the Films

The mechanical properties of the films, including the tensile strength (TS), breaking strain (BS), and toughness, were evaluated using a Texture Analyzer (Stable Micro Systems (Surrey, UK), Exponent software (Version 6.1.16.0) equipped with tensile grips (A/TG probe). The measurements followed the ASTM D882 standard method [23]. Rectangular film samples (15 mm × 70 mm) were attached to the tensile grips with an initial gauge length of 40 mm. The tests were conducted using a maximum recommended tensile load of 50 kg and a crosshead speed of 1.00 mm/s during the test and 10.00 mm/s in the post-test phase.

#### 2.7.8. Fourier Transform Infrared (FTIR) Spectroscopy of the Films

The functional groups of the samples were analyzed using a PerkinElmer Spectrum Two spectrometer (Waltham, MA, USA) with an ATR module. The FT-IR spectra were recorded in the 500–4000 cm^−1^ range with a resolution of 4 cm^−1^ for 4 scans, ensuring detailed spectral analysis.

#### 2.7.9. Structural Analysis of Films

The surface structure of the films was analyzed using scanning electron microscopy (SEM) with a HITACHI SU1510 (Tokyo, Japan). Images were taken to examine the top surface at 100× and 1000× magnification and the bottom surface at 1000× magnification, providing a detailed view of the morphology of the films.

#### 2.7.10. Statistical Analysis

All experiments were performed in triplicate, and differences between film samples were evaluated using one-way analysis of variance (ANOVA) followed by Tukey’s multiple range test for comparison. Statistical analysis was carried out using the SPSS 18.0 software (SPSS Inc., Chicago, IL, USA), with the significance set at a 95% confidence level.

## 3. Results and Discussion

### 3.1. Proximate Composition of Anchovy Byproduct and the Anchovy Byproduct Protein Hydrolysate

Anchovy byproduct was found to contain 13.92 ± 0.72% protein, 7.15 ± 0.24% lipid, 4.45 ± 0.19% ash, and 74.48 ± 0.87% moisture. After enzymatic hydrolysis, the resulting protein hydrolysate showed a significantly higher protein content (80.7 ± 0.94%), along with 6.35 ± 0.37% lipid and 12.85 ± 0.12% ash. Hydrolyzation followed by lyophilization noticeably increased the protein content while lowering the moisture and lipid levels in the final product. These changes suggest that enzymatic hydrolysis contributed to concentrating the protein fraction of anchovy waste by breaking down complex proteins into smaller, more soluble peptides. The rise in ash content may reflect the concentration of minerals during the hydrolyzation and lyophilization processes.

### 3.2. Degree of Hydrolysis

It was determined that the degree of hydrolysis in the protein hydrolysates obtained from anchovy byproduct using Flavourzyme ranged from 54% to 57%. In a study done by He, et al. [24], anchovy proteins were hydrolyzed using both endogenous enzymes and commercial proteases, including Flavourzyme and Alcalase 2.4L. The hydrolysis process resulted in a degree of hydrolysis of 33.2%. Our results indicate a good degree of hydrolysis (54–57%), suggesting that the enzymatic treatment was effective in breaking down anchovy waste proteins into smaller peptides.

### 3.3. Amino Acid Composition of Anchovy Byproduct Protein Hydrolysates

The amino acid composition of the ABPH is presented in Table 1. The total amino acid content was measured as 33.35 g/100 g sample, indicating a high release of amino acids through enzymatic hydrolysis. Among the amino acids, glutamic acid (8.84 ± 0.11 g/100 g) and aspartic acid (7.01 ± 0.51 g/100 g) were predominant, followed by serine, threonine, and proline. This profile reflects the typical amino acid distribution of fish-derived hydrolysates, where acidic amino acids are generally abundant due to their structural role in myofibrillar and sarcoplasmic proteins [25,26]. These findings are consistent with previously reported data on anchovy protein hydrolysates. Tütüncü [26] reported a total amino acid content of 32.15 g/100 g in hydrolysates obtained from anchovy byproducts, with glutamic acid (8.72 g/100 g) and aspartic acid (6.98 g/100 g) being the most abundant amino acids, in close alignment with the present results. Similarly, Korkmaz and Tokur [27] found that glutamic and aspartic acids predominated in hydrolysates produced from anchovy, whiting, and trout byproducts, and reported high levels of essential amino acids, particularly lysine, methionine, and histidine, in Flavourzyme-treated samples, which aligns with the amino acid profile obtained in this study. Pires, Leitão, Sapatinha, Gonçalves, Oliveira, Nunes, Teixeira, Mendes, Camacho, Machado, Pintado, Ribeiro, Vieira, Delerue-Matos, Lourenço, and Marques [25] characterized hydrolysates from salmon heads and hake trimmings and reported high levels of glutamic acid and threonine, confirming the recurring dominance of acidic and polar amino acids in fish-derived hydrolysates across species.

### 3.4. Film Characterization and Visual Evaluation

**Figure 1:**
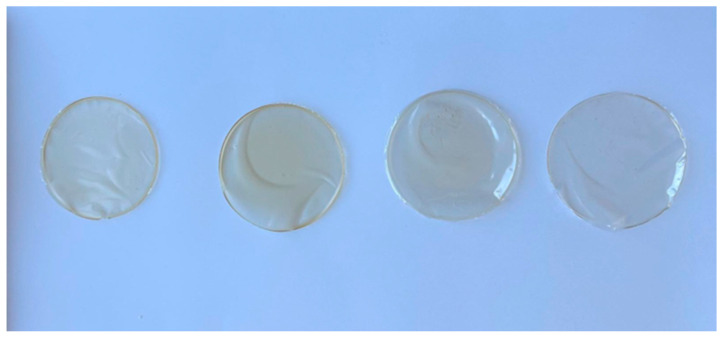
Images of film samples, from right to left. CH, CH-FPH1, CH-FPH2, and CH-FPH3.

The films produced demonstrated good integration and compatibility between the chitosan matrix and the ABPH (1%, 2%, and 3%) across all tested concentrations. The resulting films were smooth, uniform, and easy to handle, indicating effective dispersion of components. No visible signs of phase separation or surface irregularities were observed in the films by the naked eye; however, further morphological evaluation was conducted using SEM to investigate microstructural features. Visual appearances of the films under different formulation conditions are presented in Figure 1.

### 3.5. Color

The color results are presented in Table 2. The L* parameter represents brightness, with values ranging from 0 (black) to 100 (white). The a* parameter indicates color tones from green (negative values) to red (positive values), while the b* parameter reflects hues from blue (negative values) to yellow (positive values). The L* values of the films ranged from 29.38 to 33.50. The control film (CH) exhibited the highest lightness (33.50 ± 1.24), while CH-FPH1 showed the lowest value (29.38 ± 1.15), indicating a significant reduction (*p* < 0.05). Films containing higher concentrations of ABPH (CH-FPH2 and CH-FPH3) demonstrated L* values statistically similar to the control (*p* > 0.05). The slight variations in lightness can be attributed to the incorporation of hydrolysate, which may affect the color properties depending on its concentration and pigment content. In a study by Venkatachalam and Lekjing [28], chitosan-based edible films incorporated with clove essential oil and nisin exhibited L* values between 53.47 and 67.58, which are considerably higher than the L* values observed in the current study (29.38 to 33.50). This difference may be attributed to the use of essential oils and nisin, resulting in lighter-colored films due to their intrinsic color and the possible light-scattering effect in the film matrix.

The a* values ranged from 0.03 ± 0.04 to −0.66 ± 0.16, indicating a shift toward a greener shade with certain treatments. However, no statistically significant differences were observed among the samples (*p* > 0.05). Similarly, de Morais Lima, Bianchini, Guerra Dias, da Rosa Zavareze, Prentice, and da Silveira Moreira [4] reported negative a* values (−2.02 to −1.87) for chitosan–xanthan gum films with fish protein hydrolysate. The b* values of the films ranged from −0.37 to 1.29, shifting from slightly bluish to mildly yellowish tones. The control and lower concentration films had negative or near-zero b* values, giving them a bluish or neutral appearance, while the higher concentration films had significantly higher b* values (*p* < 0.05), indicating a move toward a more yellowish color.

The color difference (Δ*E*) was calculated to assess the visibility of color changes relative to the reference film (CH), which exhibited L*, a*, and b* values of 33.50, 0.03, and −0.37, respectively. A Δ*E* value exceeding three is generally considered the threshold for perceptible color change to the human eye. Among the tested samples, only the CH-FPH1 film displayed a Δ*E* value above this threshold (Δ*E* = 4.13), indicating a noticeable color variation. In contrast, CH-FPH2 (Δ*E* = 1.90) and CH-FPH3 (Δ*E* = 1.69) remained below this level, suggesting that their color differences were not visually distinguishable.

### 3.6. Moisture Content, Thickness, Swelling Ratio, and Water Solubility

The moisture content, thickness, swelling ratio, and water solubility of the films produced are summarized in Table 3. The moisture content of the films ranged from 13.13% to 17.17%. The chitosan film (CH) and films incorporated with fish protein hydrolysate at lower concentrations (CH-FPH1 and CH-FPH2) exhibited similar moisture levels, showing no statistically significant differences (*p* > 0.05). However, the film with the highest hydrolysate concentration (3%) (CH-FPH3) showed a significantly lower moisture content (13.13 ± 0.28%, *p* < 0.05), which may be attributed to the increased solid content or enhanced intermolecular interactions reducing water retention. Including ABPH showed an increase in the swelling ratio of films. While the CH-FPH3 film showed the greatest value (28.32 ± 1.06), the control film (CH) showed the lowest swelling ratio (1.75 ± 0.16). Suggesting that the presence of hydrolysates improved the water absorption capacity, probably due to their hydrophilic character and a looser polymer network structure, each increase in ABPH concentration resulted in a statistically significant rise in swelling ratio (*p* < 0.05). Apriliyani [29] examined the properties of edible films composed of casein and different concentrations of chitosan. It was observed that elevating the chitosan concentration resulted in a reduction of both moisture content and swelling degree. The film with the highest chitosan concentration had a moisture content of 29.24% and a swelling degree of 5.48%. The water solubility of the films increased progressively with the addition of ABPH. The control film (CH) exhibited the lowest solubility (21.55 ± 0.45%), while the CH-FPH3 film showed the highest value (28.47 ± 0.78%). Statistically significant differences (*p* < 0.05) were observed among most formulations, except between CH and CH-FPH1. The gradual increase in solubility may be attributed to the hydrophilic groups present in the ABPH, which promote higher water interaction and dissolution of the film matrix. Similarly, Tural and Turhan [30] observed that films formulated with anchovy byproduct proteins exhibited relatively high water solubility, which they attributed to the presence of moisture-attracting polar groups inherent in fish proteins.

The addition of ABPH caused slight changes in film thickness. However, statistical analysis showed that these differences were not significant (*p* > 0.05), suggesting that the hydrolysate concentration had little effect on the thickness of the films. The thickness values of the films in this study (0.105 to 0.121 mm) were similar to those reported by Wai, et al. [31], who developed chitosan–sodium caseinate composite edible films with thicknesses of 0.11 ± 0.01 mm and 0.12 ± 0.01 mm. In both cases, adding protein-based ingredients caused only slight changes in film thickness, which were not statistically significant. These results suggest that incorporating bioactive or protein-rich components at similar levels does not noticeably affect the physical structure or thickness of chitosan-based films.

### 3.7. Opacity

The opacity results are presented in Table 4. The incorporation of ABPH into the films led to a significant rise in opacity values (*p* < 0.05). The pure chitosan film (CH) demonstrated the lowest opacity (0.88 ± 0.08 mm^−1^), reflecting its high level of transparency. As the concentration of ABPH increased, opacity values progressively rose to 1.66 ± 0.22 mm^−1^, 1.88 ± 0.19 mm^−1^, and 2.35 ± 0.22 mm^−1^ for CH-FPH1, CH-FPH2, and CH-FPH3, respectively. This increase is likely due to enhanced light scattering resulting from hydrolysate particles or aggregates dispersed in the film matrix, along with potential phase separation at higher ABPH concentrations. Statistical comparisons revealed significant differences among most samples, although CH-FPH1 and CH-FPH2 did not differ significantly.

### 3.8. Mechanical Properties

The mechanical properties of the films were evaluated to determine their suitability for packaging applications. Tensile strength is a key indicator of a film’s ability to withstand mechanical stress without tearing or breaking, particularly during handling, storage, and transportation. It was measured by subjecting the films to uniaxial tension until failure, with the maximum stress sustained before breaking recorded as the tensile strength. Meanwhile, breaking strain refers to the film’s ability to stretch before rupture and was calculated as the percentage increase in length relative to its original dimension. Together, these parameters provide valuable insight into the film’s strength and flexibility [32]. The mechanical properties of the films, as shown in Table 3, were affected by the incorporation of ABPH. As the ABPH content increased, the tensile strength decreased significantly, from 18.04 ± 5.97 N/mm^2^ in the control (CH) to 6.16 ± 2.29 N/mm^2^ in CH-FPH3 (*p* < 0.05), indicating a weakening of the chitosan matrix. This trend is consistent with the results of de Morais Lima, Bianchini, Guerra Dias, da Rosa Zavareze, Prentice, and da Silveira Moreira [4], who found tensile strength was decreased by a fish protein hydrolysate addition into chitosan–xantham gum films. However, in contrast, Ali, Al-Ibresam, Al-Hatim, Al-Ali, Maslekar, Yao, and Agarwal [5] found that incorporating whey protein hydrolysate into chitosan films significantly enhanced the tensile strength, attributing the improvement to covalent crosslinking between amino groups of chitosan and carboxylic acid groups in the hydrolysate.

In contrast, the breaking strain in our study increased from 117.11 ± 10.70% in the control film to 153.25 ± 9.23% in CH-FPH3, suggesting enhanced film flexibility with increasing ABPH percentage. A similar increase in elongation at break was reported by de Morais Lima, Bianchini, Guerra Dias, da Rosa Zavareze, Prentice, and da Silveira Moreira [4], where the presence of protein-based additives was found to enhance flexibility, likely due to plasticizing effects and decreased crystallinity within the biopolymer matrix.

### 3.9. Fourier-Transform Infrared (FTIR) Spectroscopy

**Figure 2:**
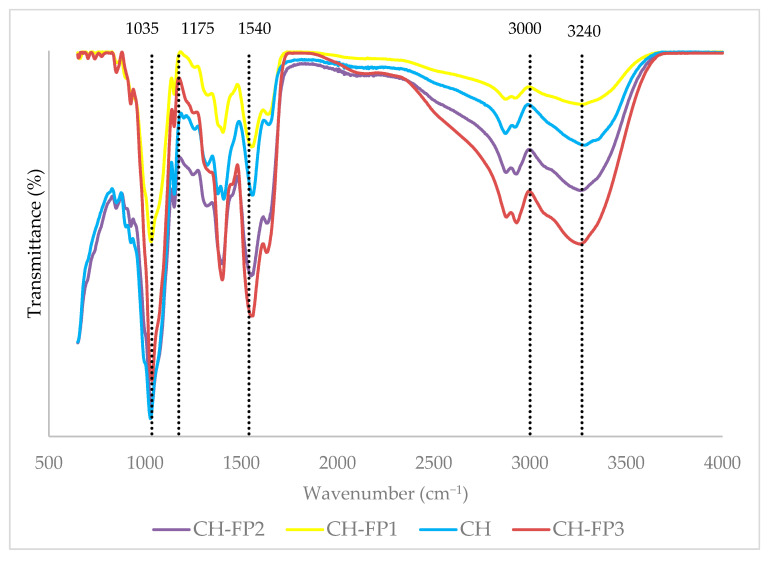
FTIR spectra of film samples.

The FTIR spectra of the chitosan (CH) films and films made with ABPH (CH-FP1, CH-FP2, and CH-FP3) are presented in Figure 2. All films showed the characteristic bands of chitosan, including a broad, intense peak around 3300 cm. The broadness and slight shift in this band upon the addition of ABPH suggest enhanced intermolecular hydrogen bonding between chitosan and peptide fragments, consistent with previous studies on chitosan-based composite films [5,33]. One of the distinctive features of the CH-FP films’ spectra is the increased intensity of the bands at ~1650 cm^−1^ and ~1540 cm^−1^, corresponding to amide I (C=O stretching) and amide II (N–H bending and C–N stretching) vibrations, respectively. The bands are typically characteristic of peptide bonds, confirming the incorporation of anchovy byproduct protein hydrolysates in the chitosan matrix. The same spectral pattern was reported by Ali, Al-Ibresam, Al-Hatim, Al-Ali, Maslekar, Yao, and Agarwal [5], whereby the incorporation of whey protein hydrolysate enhanced the amide bands of chitosan-based films.

The peaks within the range 1000–1150 cm^−1^ for C–O–C stretching of the chitosan backbone reduced slightly in intensity in CH-FP films. This may be due to the partial interaction of hydrolysate with chitosan ether groups or the change in overall molecular organization of the film. Further, the slight changes observed at ~2900 cm^−1^ (C–H stretching) across the samples also support the changes in molecular packing and chain dynamics.

The observed changes in the spectrum, especially in the amide and hydroxyl regions, reveal a good interaction and incorporation of fish protein hydrolysates and chitosan.

### 3.10. Structural Analysis

**Figure 3:**
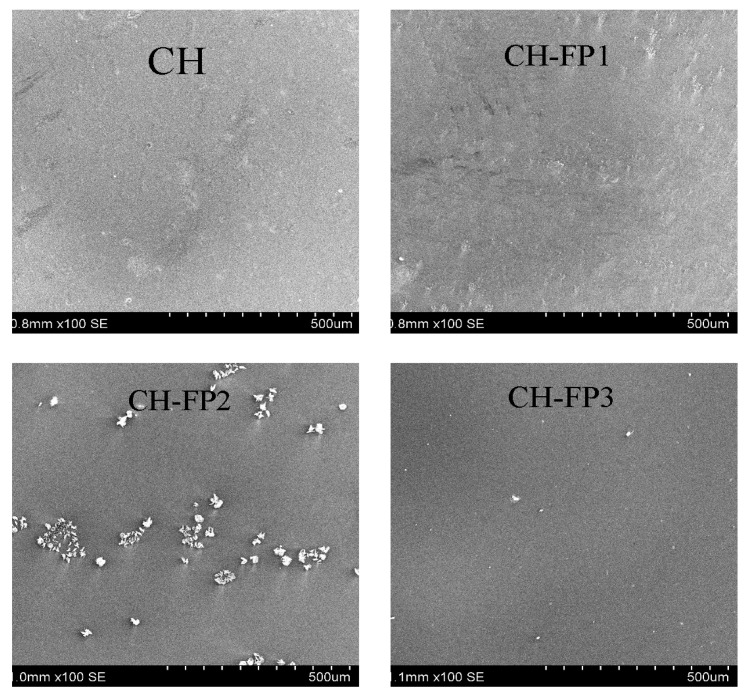
SEM micrographs of films at 100× magnification.

Figure 3 presents scanning electron microscopy (SEM) of the films that were characterized by the incorporation of increasing concentrations of ABPH. The surface of the control film (CH) was homogenous and compact, in accordance with studies on unmodified chitosan films [5]. ABPH incorporation resulted in an increase in surface roughness and the appearance of irregular aggregates with crystalline-like structures, especially observed in CH-FP2. This is either due to phase separation or limited compatibility between the hydrolysate and the chitosan matrix, similar to that observed in the chitosan/whey protein hydrolysate films reported by Ali, Al-Ibresam, Al-Hatim, Al-Ali, Maslekar, Yao, and Agarwal [5] and in the soy protein hydrolysate/chitosan films investigated by Li, et al. [34]. Remarkably, at a high hydrolysate content (CH-FP3), the surface became more uniform once more, possibly due to improved dispersion or enhanced molecular interactions, as also observed in high whey protein hydrolysate-loaded chitosan films [5].

## 4. Conclusions

This study revealed the production of films incorporating ABPH at 1%, 2%, and 3% concentrations. ABPH integration affected the physical, mechanical, and structural properties of the films. Increasing ABPH content increased film opacity, swelling capacity, and water solubility, while tensile strength decreased and elongation at break improved, indicating greater flexibility. High amide I and II band intensities and shifts in hydroxyl regions indicate ABPH incorporation and suggest hydrogen bonding and protein–chitosan interactions based on FTIR results. SEM also revealed structural changes in the film matrix with surface roughness increasing at moderate ABPH levels and uniform at a high concentration. These results indicate that ABPH could be a useful and alternative bioactive additive for biodegradable film formulations and a promising route to turn fish processing waste into value-added material for sustainable food packaging applications.

## Figures and Tables

**Table 1:** Amino acid composition of anchovy byproduct protein hydrolysates.

Amino Acids	Symbol	Amino Acid g/100 g by LC-MS/MS
Arginine	ARG	0.71 ± 0.07
Aspartic Acid	ASP	7.01 ± 0.51
Cystine	CYS	0.61 ± 0.02
Glutamic Acid	GLU	8.84 ± 0.11
Histidine	HIS	0.72 ± 0.11
Isoleucine + Leucine	ILE + LEU	1.42 ± 0.14
Lysine	LYS	1.44 ± 0.04
Methionine	MET	1.02 ± 0.04
Phenylalanine	PHE	0.62 ± 0.05
Proline	PRO	1.36 ± 0.07
Serine	SER	4.41 ± 0.03
Threonine	THR	3.56 ± 0.05
Tyrosine	TYR	0.64 ± 0.07
Valine	VAL	1.39 ± 0.12
Total		33.35

**Table 2:** Color properties of film samples.

Films	L*	a*	b*	Δ*E*
CH	33.50 ± 1.24 ^a^	0.03 ± 0.04 ^a^	−0.37 ± 0.06 ^a^	
CH-FPH1	29.38 ± 1.15 ^b^	−0.14 ± 0.06 ^a^	−0.23 ± 0.23 ^a^	4.13
CH-FPH2	32.88 ± 1.43 ^ab^	−0.65 ± 0.46 ^a^	1.29 ± 1.01 ^b^	1.90
CH-FPH3	32.71 ± 0.74 ^ab^	−0.66 ± 0.16 ^a^	0.96 ± 0.53 ^b^	1.69

Different superscript letters within the same column indicate significant differences (*p* < 0.05) according to one-way ANOVA followed by Tukey’s multiple range test.

**Table 3:** Moisture, thickness, swelling ratio, and water solubility of films.

Films	Moisture (%)	Thickness (mm)	Swelling Ratio (g/g)	Water Solubility (%)
CH	17.14 ± 0.46 ^a^	0.105 ± 0.05 ^a^	1.75 ± 0.16 ^a^	21.55 ± 0.45 ^a^
CH-FPH1	15.61 ± 1.31 ^a^	0.110 ± 0.08 ^a^	7.91 ± 0.62 ^b^	22.51 ± 0.89 ^ab^
CH-FPH2	17.17 ± 0.46 ^a^	0.112 ± 0.04 ^a^	20.31 ± 3.88 ^c^	24.47 ± 0.46 ^b^
CH-FPH3	13.13 ± 0.28 ^b^	0.121 ± 0.10 ^a^	28.32 ± 1.06 ^d^	28.47 ± 0.78 ^c^

Different superscript letters within the same column indicate significant differences (*p* < 0.05) according to one-way ANOVA followed by Tukey’s multiple range test.

**Table 4:** Optical and mechanical properties of films.

Films	Transmittance (%)	Opacity (mm^−1^)	Tensile Strength N/mm^2^	Breaking Strain (%)
CH	88.73 ± 1.50 ^a^	0.88 ± 0.08 ^a^	18.04 ± 5.97 ^a^	117.11 ± 10.70 ^a^
CH-FPH1	83.80 ± 3.12 ^b^	1.66 ± 0.22 ^b^	11.97 ± 3.48 ^ab^	125.26 ± 14.86 ^ab^
CH-FPH2	74.03 ± 1.29 ^c^	1.88 ± 0.19 ^b^	6.33 ± 0.81 ^b^	147.59 ± 13.53 ^b^
CH-FPH3	70.10 ± 2.69 ^c^	2.35 ± 0.22 ^c^	6.16 ± 2.29 ^b^	153.25 ± 9.23 ^c^

Different superscript letters within the same column indicate significant differences (*p* < 0.05) according to one-way ANOVA followed by Tukey’s multiple range test.

## Data Availability

The original contributions presented in this study are included in the article. Further inquiries can be directed to the corresponding author.
